# Biodegradable 2D Fe–Al Hydroxide for Nanocatalytic Tumor‐Dynamic Therapy with Tumor Specificity

**DOI:** 10.1002/advs.201801155

**Published:** 2018-10-09

**Authors:** Zhenbang Cao, Liang Zhang, Kang Liang, Soshan Cheong, Cyrille Boyer, J. Justin Gooding, Yu Chen, Zi Gu

**Affiliations:** ^1^ School of Chemical Engineering and Australian Centre for NanoMedicine (ACN) University of New South Wales Sydney NSW 2052 Australia; ^2^ Department of Ultrasound the First Affiliated Hospital of Chongqing Medical University Chongqing 400010 China; ^3^ Electron Microscope Unit University of New South Wales Sydney NSW 2052 Australia; ^4^ School of Chemistry ARC Centre of Excellence in Convergent Bio‐Nano Science and Technology and Australian Centre for NanoMedicine (ACN) University of New South Wales Sydney NSW 2052 Australia; ^5^ State Key Laboratory of High Performance Ceramics and Superfine Microstructure Shanghai Institute of Ceramics Chinese Academy of Sciences Shanghai 200050 China

**Keywords:** 2D nanosheets, layered double hydroxide, nanomedicine, therapeutic catalysis, tumor microenvironment responsiveness

## Abstract

Therapeutic nanocatalysis has emerged as an intriguing strategy for efficient cancer‐specific therapy, but the traditional inorganic nanocatalysts suffer from low catalytic efficiency and difficulty in biodegradation, hindering their further clinical translation. Herein, a tumor microenvironment‐triggered, biodegradable and biocompatible nanocatalyst employing 2D hydroxide nanosheet is presented, and is shown to have high catalytic capacity to efficiently produce abundant hydroxyl radicals under the tumor microenvironment and consequently kill tumor cells selectively. A polyethylene glycol (PEG)‐conjugated Fe^2+^‐containing hydroxide nanosheet is successfully constructed via a facile but efficient bottom‐up approach that concurrently realizes nanosheet synthesis and PEGylation. Importantly, the nanosheets are featured with high catalytic activity to disproportionate H_2_O_2_ in tumors, and consequently generate abundant hydroxyl radicals at a high reaction rate under tumorous acidic condition; the highly toxic hydroxyl radicals, as a result, cause the death of tumor cells in vitro and suppress the tumor growth in vivo without the use of any supplementary toxic agent, only with the biocompatible nanocatalysts. Meanwhile, the desirable biodegradation and biocompatibility of the hydroxide nanosheet render a high degree of safety to the organism. Therefore, this work provides the first paradigm of biodegradable 2D nanocatalytic platform with concurrently high catalytic‐therapeutic performance and biosafety for efficient tumor‐specific treatment.

Cancer is a life‐threatening condition where cells grow uncontrollably with a specific biological microenvironment.^1^ One of the microenvironment characteristics is mild acidity, as a result of fast metabolism of tumor cells and lactic acid overproduction.^2^ Another difference between the cancer and normal tissues is the high expression of reactive oxygen species (ROS) in tumors.^3^ ROS species encompass hydrogen peroxide (H_2_O_2_), superoxide anion radical, singlet oxygen, and hydroxyl radical (·OH), amongst which the hydroxyl radical is the most reactive ROS.^4^ ROS is usually regulated by the redox balance in cancers and serves a second messenger in cell signaling.^5^ Nevertheless, the overproduction of ROS could cause severe oxidative damage to tumor cells, thus providing a practical therapeutic approach for cancer treatment.^6^ This approach can be realized by introducing iron catalyst to cancer cells where the high level of H_2_O_2_ can be consequently disproportionated into highly reactive hydroxyl radicals via acidity‐triggered Fenton reactions.^7^ However, challenges still remain with regards to developing a Fenton catalyst with high catalytic‐therapeutic efficiency to tumors and concurrent high level of biocompatibility to normal cells.

Recently, two‐dimensional (2D) nanomaterials have attracted broad attention with excellent performance in versatile applications such as catalysis and biomedicine,^8^ owing to their remarkably high surface‐to‐volume area and a considerable number of reactive sites.^9^ Ultrathin layered double hydroxide (LDH) nanosheets are one of the most representative 2D nanomaterials, with a general formula of [M^2+^_1‐_ *_x_*M^3+^ *_x_*(OH)_2_]*^x^* ^+^ (A^n−^)*_x_*_/n_ · mH_2_O, whereby M represents the divalent and trivalent metal ions that form the hydroxide layers, and A^n−^ denotes the exchangeable anion interacted with the layers.^10^ The unique coordination structure endows LDHs with certain unique features and consequently appealing biomedical applications. For instance, the hydroxide layer of LDHs can have various metal compositions, which enables the functional metal cations to be incorporated into LDHs for molecular imaging of tumors.^11^ Moreover, the coordination of OH group for metal ions makes LDHs sensitive to the mildly acidic solutions, causing the biodegradation of LDHs to release biocompatible cations, ions, and H_2_O, which would minimize the long‐term in vivo accumulation risk as well as facilitate drug release, homogeneous catalysis and the consequent enhanced therapeutic effect.[[qv: 11b,f]] Compared with the multilayered LDH nanoparticles, ultrathin LDH nanosheets demonstrate a high level of drug loading efficiency and catalytic functions.^12^ However, one of the major obstacles in the broad bioapplications of LDH nanosheets is that the conventional exfoliation method involves the use of an organic solvent (e.g., formamide) which is used to generate and also maintain the exfoliation status but harmful to the biological systems.

Herein, we report, for the time, on the construction of a polyethylene glycol (PEG)‐conjugated, ferrous ion‐containing 2D LDH monolayer nanosheets (designated as PEG/Fe‐LDH) via a novel and efficient solvent‐free bottom‐up method, toward high catalytic‐therapeutic efficiency specifically in tumors. These 2D PEG/Fe‐LDH nanosheets are featured with a unique planar topology with the thickness of ≈2.4 nm, a lateral size of ≈198 nm, and high level of colloidal stability in saline, as a result of efficient delamination and PEGylation during the facile synthetic procedure. Importantly, the PEG/Fe‐LDH nanosheets are capable of catalyzed production of a considerable amount of hydroxyl radicals via pH‐responsive and H_2_O_2_‐triggered Fenton reaction, which have shown high catalytic efficiency as evidenced by Michaelis–Menten constant *K*_m_ = 0.09 × 10^−3^ m and maximum reaction velocity *V*_max_ = 1.76 × 10^−6^ m s^−1^. The origin of hydroxyl radical generation induced by the PEG/Fe‐LDH was further investigated under different pH environments. In mildly acidic conditions, iron on the nanosheet surface together with released iron ions from the nanosheet attribute to hydroxyl radical generation. In vitro and in vivo assessment performed in breast cancer cell culture and tumor xenografted Balb/c mice showed that the PEG/Fe‐LDH nanocatalysts can serve as an efficient catalytic therapeutic agent. Specially, the PEG/Fe‐LDH treatment exhibited as low as 23% cell viability at the extremely low dose of 6 µg mL^−1^ in a 4T1 cell culture that mimics the tumor microenvironment. The intratumoral administration of PEG/Fe‐LDH nanocatalysts showed 59% tumor suppression rate in the tumor‐bearing mice without the use of any naturally toxic agent, only with the triggering of catalytic Fenton reaction in tumor by PEG/Fe‐LDHs nanocatalysts. Moreover, the intravenous administration of biodegradable PEG/Fe‐LDHs at the dose up to 100 mg kg^−1^ Fe was demonstrated to induce no obvious damage to the major organ tissues. Therefore, these 2D PEG/Fe‐LDH nanocatalysts could be a promising Fenton catalytic agent to achieve high therapeutic efficiency on combating tumors while exhibit desired biocompatibility in normal tissues.

**Figure** **1** illustrates the procedure for the synthesis of 2D PEG/Fe‐LDH nanosheets as a therapeutic nanocatalyst. The PEG/Fe‐LDH nanosheets were synthesized via a facile solvent‐free bottom‐up method, in which ferrous and aluminum ions were co‐precipitated with sodium hydroxide at a constant pH value, immediately followed by introducing phosphonic acid terminated PEG molecules and then a hydrothermal treatment. This facile three‐step synthesis method realizes the fabrication of delaminated LDH nanosheet and simultaneously achieves PEG molecule conjugation. X‐ray diffraction (XRD) pattern of the Fe‐LDH nanoparticles showed characteristic (00*l*) diffraction peaks with (003) diffraction peak at 2θ = 11.68° (**Figure** **2**a). In comparison, the PEG/Fe‐LDH nanosheet did not display any obvious (00*l*) diffraction peaks (Figure 2a), indicating the lack of a long‐range ordered structure.^13^ The delamination status of PEG/Fe‐LDH was further confirmed by atomic force microscopy (AFM). The average thickness of PEG/Fe‐LDH nanosheets was measured to be ≈2.4 nm by AFM (Figure 2b,c). In the very thin film spots, the average thickness was ≈1.2 nm (Figure S1, Supporting Information). These results indicate that each nanosheet encompasses one single hydroxide layer (≈1.2 nm) and PEG coating on the nanosheet surface (≈1.2 nm). The delamination of nanosheets via the solvent‐free bottom‐up method generated the similar thickness of the LDH that was exfoliated by formamide solvent.[[qv: 12b]]

**Figure 1:**
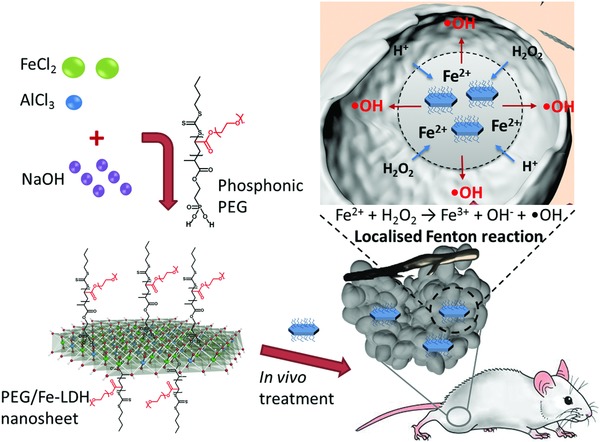
Synthetic procedure, microstructure, and therapeutic‐catalytic function of 2D PEG/Fe‐LDH nanosheets.

**Figure 2:**
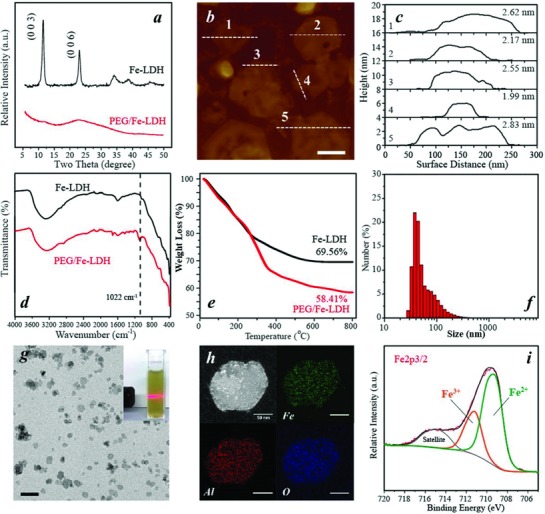
Physicochemical structure of PEG/Fe‐LDH nanosheets and Fe‐LDH nanoparticles. a) XRD patterns. b) AFM image of PEG/Fe‐LDHs, scale bar = 100 nm. c) Thickness of PEG/Fe‐LDHs measured from AFM image. d) FTIR spectra. e) TGA profiles. f) Size distribution of PEG/Fe‐LDHs via DLS. g) TEM image of PEG/Fe‐LDHs, scale bar = 500 nm (insert: digital photo of PEG/Fe‐LDH colloidal suspension). h) STEM image of PEG/Fe‐LDH nanosheet and corresponding elemental mapping of Fe, Al, and O; scale bar = 50 nm. i) Fe 2p 3/2 XPS spectrum of PEG/Fe‐LDHs.

Fourier‐transform infrared spectroscopy (FTIR) and thermal gravimetric analysis (TGA) were used to examine the PEG composition on LDH nanosheets. The stretching vibration of C—O from PEG molecules was recorded at 1100 cm^−1^ in the PEG/Fe‐LDH spectrum but not in the Fe‐LDH spectrum (Figure 2d),^14^ implying the successful PEG attachment on the PEG/Fe‐LDH. From TGA results, the difference in the mass loss between PEG/Fe‐LDHs and Fe‐LDHs indicated that the proportion of PEG on the PEG/Fe‐LDH was ≈11.2% (Figure 2e), and the grafting density of PEG was calculated to be ≈0.02 PEG chain nm^−2^ of LDH surface. The PEG/Fe‐LDH exhibited well‐dispersed colloidal suspension (Figure 2f), with 198 nm in average hydrodynamic size, 0.2 in polydispersity index and +18.8 mV in zeta potential. Importantly, the size distribution and average size of PEG/Fe‐LDHs in saline remained the same as that in water (Figure S2a, Supporting Information). In comparison, the Fe‐LDH nanoparticles were aggregated in water or saline with the large particle size beyond the DLS measurement limitation (Figure S2b, Supporting Information). The improved colloidal stability could be attributed to the in situ PEG conjugation that endows the nanosheets with steric repulsion.^15^ Transmission electron microscopy (TEM) image of PEG/Fe‐LDH showed the typical hexagonal morphology of LDH with an average lateral size around 150 nm (Figure 2g).^16^ Energy‐dispersive X‐ray spectroscopy elemental mapping (EDS‐mapping) under scanning‐TEM (STEM) mode and corresponding X‐ray spectrum of a PEG/Fe‐LDH nanosheet revealed the coexistence and homogenous distribution of Fe, Al, O signals across the nanosheet (Figure 2h; Figure S3, Supporting Information). Inductively coupled plasma mass spectrometry revealed that the molar ratio of Fe to Al in the PEG/Fe‐LDH was ≈2:1, and the mass proportion of Fe in PEG/Fe‐LDH was ≈34.6%, being close to the designed ratio. From X‐ray photoelectron spectroscopy (XPS) spectrum, the peak fitting analysis of Fe 2p in PEG/Fe‐LDH identified two distinct iron species which were Fe^2+^ (2p_3/2_ peak at 709.1 eV) and Fe^3+^ (2p_3/2_ peak at 711.2 eV).^17^ The amount of iron at low oxidation status (Fe^2+^) was estimated to be 70% of the total iron (Figure 2i). The high content of ferrous ions in the nanosheet could facilitate the Fenton‐reaction based catalytic activity of PEG/Fe‐LDHs.^18^

The efficiency of radical generation from H_2_O_2_ decomposition by using the PEG/Fe‐LDH as a Fenton nanocatalyst was evaluated via 3,3′,5,5′‐tetramethylbenzidine (TMB) assay (Figure S4, Supporting Information). The intermediate ·OH generated during H_2_O_2_ decomposition can oxidize TMB to a blue product with light absorption λ_max_ 650 nm (Figure S5, Supporting Information). The PEG/Fe‐LDH has catalytic activity dependent on catalyst concentration, pH, temperature, and substrate H_2_O_2_ concentration (**Figure** **3**a–d), which was also found in other Fenton reaction catalysts.^19^ The optimal conditions (37 °C, pH = 4) of the TMB assay were adopted in the following kinetics analysis. The absorbance at λ650 nm was plotted against time with addition of H_2_O_2_ at different concentrations into PEG/Fe‐LDHs (Figure 3d), and the initial velocities were calculated. The initial velocities against H_2_O_2_ concentration were fitted into the Michaelis–Menten equation to determine the Michaelis–Menten constant (*K*_m_) and maximum velocity (*V*_max_) (Figure 3e). The *K*_m_ value of PEG/Fe‐LDHs was calculated to be 0.09 × 10^−3^ m, indicating that the PEG/Fe‐LDH nanocatalyst could achieve 50% of maximum catalytic activity at the H_2_O_2_ concentration as low as 0.09 × 10^−3^ m. Importantly, the low *K*_m_ value of PEG/Fe‐LDHs indicates that the catalytic activity of PEG/Fe‐LDHs could be sufficiently high to exhibit their therapeutic function in cancers, considering the endogenous H_2_O_2_ concentration in the tumor microenvironment usually below 0.1 × 10^−3^ m. In comparison, the *K*_m_ of Fe‐LDHs was 0.16 × 10^−3^ m, being higher than that of PEG/Fe‐LDHs. The *V*_max_ of PEG/Fe‐LDHs indicates that the PEG/Fe‐LDHs could catalyze H_2_O_2_ at the maximum velocity of 1.76 × 10^−6^ m s^−1^, which is greater than the *V*_max_ value of Fe‐LDHs (1.47 × 10^−6^ m s^−1^). The relatively low *K*_m_ value and high *V*_max_ value of PEG/Fe‐LDHs compared with Fe‐LDHs could be attributed to their larger surface‐to‐volume ratio as a result of delamination; the larger surface area renders the LDH catalyst more active catalytic sites and thus markedly increases the catalytic activity.

**Figure 3:**
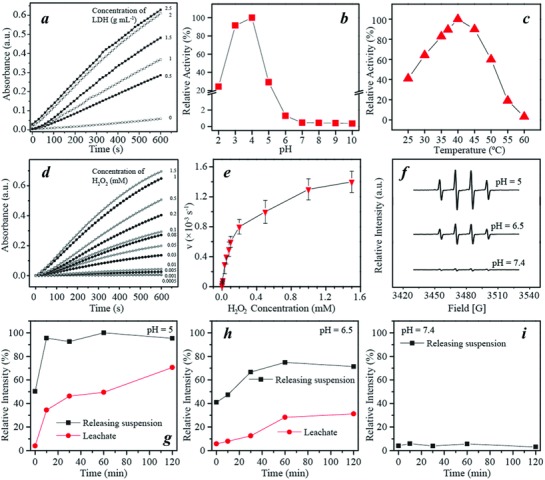
Catalytic activity of PEG/Fe‐LDHs. Relative activity of PEG/Fe‐LDH and H_2_O_2_ system with different a) LDH concentrations, b) pH values, and c) temperature. d) Absorbance of PEG/Fe‐LDH with the addition of varied concentration of H_2_O_2_, measured at λ = 650 nm via TMB assay. e) Michaelis–Menten steady‐state kinetics by plotting reaction velocity (*v*) against H_2_O_2_ concentration. f) ESR spectra of PEG/Fe‐LDHs with addition of H_2_O_2_ in the buffers of varied pH values. g–i) Relative intensity of ESR signal induced from releasing suspension and leachate at (g) pH 5.0, (h) pH 6.5, and (i) pH 7.4.

To verify the radical species from the catalytic reaction, electron spin resonance (ESR) spectroscopy was applied by using 5,5‐dimethyl‐1‐pyrroline *N*‐oxide as a spin trap. The addition of H_2_O_2_ to the PEG/Fe‐LDH suspension under the mildly acidic condition (pH 6.5 and 5.0) generated a considerable amount of hydroxyl radicals, as shown in ESR spectra with characteristic 1:2:2:1 hydroxyl radical signals (Figure 3f).^20^ Interestingly, this obvious signal was not observed in the ESR spectrum under the neutral pH environment (Figure 3f; Figure S6, Supporting Information). This acidity‐triggered hydroxyl radical generation implies the high selectivity and specificity of the PEG/Fe‐LDH as a therapeutic nanocatalyst to treat cancers under the mildly acidic tumor microenvironment.

ESR spectroscopy was used to further explore the origin of hydroxyl radical generation induced by the PEG/Fe‐LDH nanosheets under different pH conditions. The PEG/Fe‐LDH nanosheets were dialyzed or suspended in the buffers of pH 5.0, 6.5, and 7.4 for 2 h. The leachate or the suspension were subsequently collected from two releasing solutions, respectively, and mixed with H_2_O_2_ for ESR analysis. In the buffers of pH 5.0 and 6.5, the ESR signal intensity of hydroxyl radicals generated from leachate increased during the 2 h release period, with its proportion to the signal intensity induced by the whole releasing suspension increased from 34% at 10 min to 71% at 2 h (Figure 3g). This result corresponds to the percentage of iron released in the buffer of pH 5.0 where the released iron was 7% of the total iron after 10 min and raised to 59% after 2 h (Figure S7, Supporting Information), confirming that the ESR signal of hydroxyl radicals in the leachate is strongly correlated with the Fe^2+^ and Fe^3+^ ions released from the nanosheet. Similarly, under pH 6.5, the hydroxyl radicals generated from the Fe^2+^ and Fe^3+^ ions were 8% and 31% of that from the suspension after 10 min and 2 h release, respectively (Figure 3h; Figure S7, Supporting Information). By contrast, there was no obvious iron released within 2 h in the buffer of pH 7.4 (Figure S7, Supporting Information), and the ESR signal from the leachate or the suspension was barely detected (Figure 3i). To sum up the aforementioned results from the ESR and the associated elemental analysis, it can be concluded that (1) homogenous and heterogeneous Fenton reactions coexist at mildly acidic conditions, mediated by the released irons and surface irons of the PEG/Fe‐LDH nanosheet, respectively; (2) heterogeneous catalysis dominates the early stage of the reaction, but with increased Fe^2+^ and Fe^3+^ release from the nanosheet, homogeneous catalysis becomes a vital process; and (3) the generation of hydroxyl radicals from the nanosheet under the neutral pH environment is negligible.

To further investigate the biodegradation of the PEG/Fe‐LDH nanosheet in the tumor‐specific acidic microenvironments, the morphology and structure evolution of the nanosheets was visualized by TEM after suspending the nanosheets in a buffer solution of pH 5.0 for a certain period of time (**Figure** **4**). The nanosheet appeared intact within the first 10 min (Figure 4a,e). Afterward, the nanosheets gradually dissolved from the center toward the edge. At 2 h only a few particles could be observed with an “O‐Ring” morphology (Figure 4d). This “center‐to‐edge” disintegration behavior could be attributed to the PEG surface modification. Due to the relatively large size of PEG compared to the interlayer gallery distance, the PEG attached on the edge of the nanosheets, which can be clearly observed under TEM (Figure 4h), can block the interlayer space, thus protecting the edge of the nanosheets from collapsing within 2 h dissolution period. Meanwhile, H^+^ ions penetrate into the interlayer space and enable the OH group at the nanosheet center easily to be protonated.^21^ At 4 h, there were no particles that could be observed (Figure S8, Supporting Information), indicating the further disintegration of the nanosheet edge and the consequent complete nanosheet collapse. The biodegradable behavior of PEG/Fe‐LDHs is beneficial for the homogenous Fenton reaction activity, which also serves an advantageous property that contributes to the biocompatibility of a bioapplied nanomaterial.^22^

**Figure 4:**
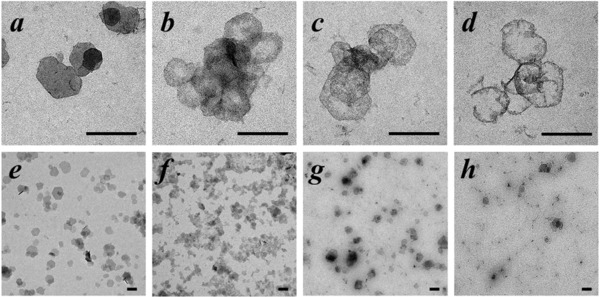
Biodegradability assay. Biodegradation performance of PEG/Fe‐LDH nanosheets in a buffer of pH 5.0 for a,e) 10 min, b,f) 30 min, c,g) 60 min, and d,h) 2 h; scale bar = 200 nm.

The in vitro anticancer function induced by PEG/Fe‐LDHs was investigated in a 4T1 cell culture under conditions that mimic the tumor microenvironment. A cell culture medium of pH 6.5 with addition of low concentration of H_2_O_2_ was used to simulate the tumor microenvironment, which showed negligible influence on the cell viability in comparison with the growth medium of pH 7.4 (Figure S9, Supporting Information). When the PEG/Fe‐LDHs were used to treat 4T1 cells, there was a significant nanosheet dose‐dependent cytotoxicity at pH 6.5 (**Figure** **5**a). The reduction in cell viability after 24 h incubation of 6 µg mL^−1^ PEG/Fe‐LDHs amounted to 77% at pH 6.5, while no obvious change in cell viability was observed at pH 7.4 (Figure 5a,b). The cellular uptake of PEG/Fe‐LDHs exhibited the same time‐course trend and slightly higher internalization efficiency compared with Mg‐LDHs reported previously.^23^ The PEG/Fe‐LDH nanosheets were internalized efficiently by cells with 48% internalized after 24 h, which was significantly higher than the Fe‐LDH nanoparticles (Figure 5c,d; Figure S10, Supporting Information), probably because of the improved colloidal stability of the PEG/Fe‐LDHs. When 2′,7′‐dichlorofluorescein diacetate (DCFH‐DA) was employed as a fluorescence ROS probe, the cells displayed strong green fluorescence with PEG/Fe‐LDHs and H_2_O_2_ treatment at pH 6.5 (Figure 5h). However, a low level of fluorescence signal was observed in the cells with the control treatments (i.e., PEG/Fe‐LDH only at pH 6.5, H_2_O_2_ only at pH 6.5, or PEG/Fe‐LDH and H_2_O_2_ at pH 7.4) (Figure 5e–g), indicating the H_2_O_2_‐triggered, pH‐responsive intracellular hydroxyl radical generation.

**Figure 5:**
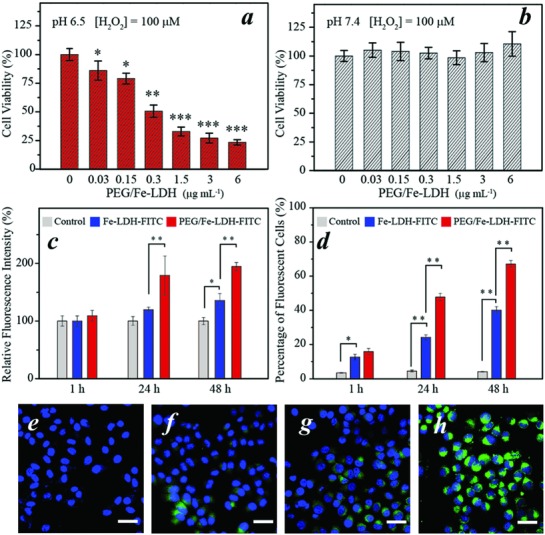
In vitro catalytic therapeutic activity of PEG/Fe‐LDHs. a,b) Cytotoxicity of PEG/Fe‐LDHs to 4T1 cells in the presence of 100 × 10^−6^ m H_2_O_2_ at (a) pH 6.5 and (b) pH 7.4. c,d) Cellular uptake of Fe‐LDH‐FITC and PEG/Fe‐LDH‐FITC monitored by flow cytometry, represented by c) relative intensity and d) percentage of fluorescent cells after a certain period of time. e–h) Confocal images of DCFH‐DA and DAPI stained 4T1 cells treated with (e) PEG/Fe‐LDH only, (f) H_2_O_2_ only, both PEG/Fe‐LDHs and H_2_O_2_ at (g) pH 7.4 and (h) pH 6.5; scale bar = 50 µm. Data are presented as mean ± SD; *P < 0.05, **P < 0.01, and ***P < 0.001.

Prior to the in vivo therapeutic assessment, the biosafety performance of PEG/Fe‐LDHs was evaluated in both normal cells (Hs27 fibroblast cells) and healthy Balb/c mice. The treatment of fibroblasts (Hs27 cells) with 0–12 µg mL^−1^ PEG/Fe‐LDH showed no influence on cell viability (Figure S11, Supporting Information), signifying the biocompatibility of PEG/Fe‐LDH to normal cells. To evaluate the in vivo biosafety, Balb/c mice were administered intravenously with PEG/Fe‐LDHs at a low dose of 10 mg kg^−1^ Fe, a medium dose of 40 mg kg^−1^ Fe and a high dose of 100 mg kg^−1^ Fe. During the treatment period of 30 days, the mice were all at stable growth rate with no significant difference between the control group and treatment groups (**Figure** **6**a). The blood of mice was collected after a 30‐day treatment period for biochemical indexes and blood cells measurement including alanine transaminase, creatinine kinase, aspartate transaminase, creatinine, blood urea nitrogen, lactate dehydrogenase (LDH‐H), total bilirubin, white blood cells, red blood cells, hemoglobin, hematocrit, mean corpuscular hemoglobin (MCH), MCH concentration, platelets, and mean corpuscular volume (Figure S12, Supporting Information). All the indexes with PEG/Fe‐LDH treatment exhibited no significant variation in comparison to the control group (Figure S12, Supporting Information), indicating that the PEG/Fe‐LDH at the high dose up to 100 mg kg^−1^ Fe has little impact on the blood biochemical status and no interference with the kidney and liver functions. The histopathological images of major organs (i.e., heart, liver, spleen, lung, and kidney) with the PEG/Fe‐LDH treatment showed no observable pathological abnormalities (Figure 6b), indicating the high level of histocompatibility of PEG/Fe‐LDHs. The results from the above biosafety evaluation suggest the high biocompatibility of PEG/Fe‐LDHs, guaranteeing the further potential in vivo therapeutic applications.

**Figure 6:**
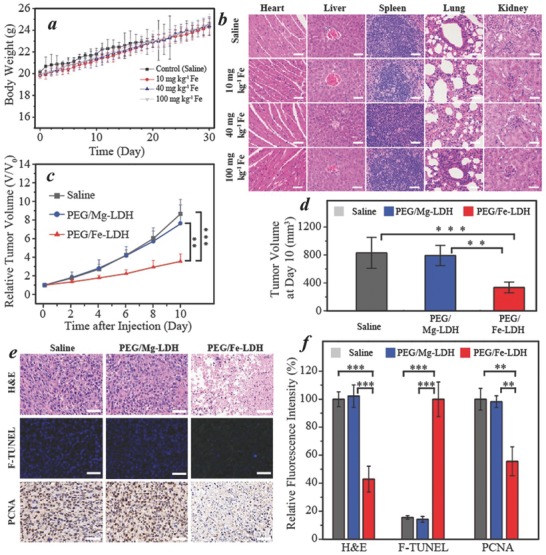
In vivo biosafety assessment and catalytic therapeutic performance of the PEG/Fe‐LDH nanocatalyst. a) Body weights of Balb/c mice after intravenous injection of saline and PEG/Fe‐LDHs (10, 40, and 100 mg kg^−1^ Fe). b) Histological images of the major organs (heart, liver, spleen, lung, and kidney) collected on day 30 after intravenous injection of saline and PEG/Fe‐LDHs; scale bar = 50 µm. c) Relative tumor volume of 4T1 tumor‐bearing Balb/c mice with intratumoral treatment of saline, PEG/Mg‐LDHs and PEG/Fe‐LDHs. d) Tumor volume on day 10 with different treatments. e) Histopathological images and f) the corresponding fluorescence intensity of the dissected tumor tissues; scale bar = 50 µm. Data are presented as mean ± SD; *P < 0.05, **P < 0.01, and ***P < 0.001.

Encouraged by the desirable in vitro catalytic therapeutic performance and high biocompatibility of PEG/Fe‐LDH nanosheets, the in vivo anticancer function was further assessed by intratumoral administration of PEG/Fe‐LDHs into 4T1 tumor‐xenografted Balb/c mice. All animal experiment operations were performed with approval of the Animal Ethics Committees of University of New South Wales and Chongqing Medical University. The PEG/Mg‐LDH nanoparticles with equivalent dose and saline were applied as controls. As shown in Figure 6c,d, significant tumor growth inhibition was achieved in the PEG/Fe‐LDH group with the relative tumor volume being 41% and 47% of those in the saline and PEG/Mg‐LDH treatment respectively. Such significant therapeutic performance was attributed to the efficient interaction between the PEG/Fe‐LDH nanocatalyst and intratumoral H_2_O_2_, thus triggering a localized Fenton reaction accompanied with ·OH species generation and subsequently tumor cell damage. In comparison, the tumor inhibition induced by PEG/Mg‐LDHs was negligible, which presented a similar tumor volume with the saline group at each time point, indicating that the iron component within LDH plays an indispensable role in therapeutic catalysis‐induced tumor inhibition.

To further confirm the anticancer effect and mechanism of the PEG/Fe‐LDH nanocatalyst, the pathological damages of the tumors caused by PEG/Fe‐LDHs were evaluated by histopathological studies of the dissected tumor tissues (Figure 6e,f). In the hematoxylin and eosin staining images, a large number of the destructed cells were observed in the PEG/Fe‐LDH group, but not shown in the control groups (i.e., saline and PEG/Mg‐LDH). The terminal deoxynucleotidyl transferase dUTP nick end labeling (TUNEL) evaluation showed green‐fluorescent apoptosis‐positive cells in the PEG/Fe‐LDH group but not in the control groups. To be more specific, the green fluorescence intensity in the PEG/Fe‐LDH was fivefold higher than that in the saline and PEG/Mg‐LDH treatment, demonstrating significant apoptosis in the cells treated with PEG/Fe‐LDHs (Figure 6f). Immunochemical staining of proliferating cell nuclear antigen of tumor tissues indicated the in vivo cell proliferation activities in which the number of PCNA‐positive cells was significantly lower in the PEG/Fe‐LDH group compared with the controls.

In summary, this work presents a biodegradable and biocompatible 2D iron‐containing LDH nanosheet as a new therapeutic nanocatalyst for efficient tumor inhibition by triggering the localized catalytic Fenton reaction in response to the specific tumor microenvironment. A facile but efficient solvent‐free exfoliation method was developed to fabricate PEGylated monolayer LDH nanosheets with high colloidal stability. These PEG/Fe‐LDH nanocatalysts exhibited high catalytic efficiency of Fenton reaction to generate abundant hydroxyl radicals under specific stimuli of the tumor microenvironment. The overproduced hydroxyl radicals were demonstrated to be induced by iron from the surface of nanosheets (heterogeneous reaction) and also from the released ions (homogeneous reaction). The homogeneous reaction played a dominant role under mildly acidic conditions, as a result of nanosheet biodegradation at pH values below 6.5. The catalytic reaction‐associated therapeutic performance was reflected by significant cancer cell suppression both in vitro and in vivo. The PEG/Fe‐LDH nanocatalyst‐treated 4T1 cancer cells exhibited only 23% cell viability at the extremely low dose of 6 µg mL^−1^ under the tumor microenvironment‐mimicking condition. The in vivo administration of PEG/Fe‐LDH nanocatalysts achieved 59% tumor suppression rate against 4T1 tumor‐xenografted Balb/c mice without the use of any supplementary toxic agents, while no tissue damages to major normal organs could be observed and monitored. Overall, this study introduces a biodegradable and biocompatible 2D hydroxide nanosheet that exhibits highly desired therapeutic function for selective cancer treatment under specific microenvironment triggers, and also provides a new strategy to construct 2D catalytic‐therapeutic agent for cancer‐specific therapy.

## Conflict of Interest

The authors declare no conflict of interest.

## Supporting information

Supplementary
