# Supplementary material for: Biodegradable 2D Fe–Al Hydroxide for Nanocatalytic Tumor‐Dynamic Therapy with Tumor Specificity
Source: Adv Sci (Weinh). 2018 Oct 9;5(11):1801155. doi: 10.1002/advs.201801155 (PMC6247031; doi:10.1002/advs.201801155)
Supplement: Supplementary file 1 — Supplementary [file ADVS-5-1801155-s001.pdf]

Copyright WILEY-VCH Verlag GmbH & Co. KGaA, 69469 Weinheim, Germany, 2018.

ADVANCED SCIENCE
Open Access

Supporting Information

for *Adv. Sci.,* DOI: 10.1002/advs.201801155

Biodegradable 2D Fe–Al Hydroxide for Nanocatalytic Tumor-Dynamic Therapy with Tumor Specificity

Zhenbang Cao, Liang Zhang, Kang Liang, Soshan Cheong, Cyrille Boyer, J. Justin Gooding, Yu Chen, and Zi Gu**

Supporting Information

Biodegradable 2D Fe-Al Hydroxide for Nanocatalytic Tumor-Dynamic Therapy with Tumor Specificity

Zhenbang Cao, Liang Zhang, Kang Liang, Soshan Cheong, Cyrille Boyer, J. Justin Gooding, Yu Chen and Zi Gu**

Z. Cao, Dr. K. Liang, Prof. C. Boyer, Dr. Z. Gu
School of Chemical Engineering and Australian Centre for NanoMedicine (ACN), University of New South Wales, Sydney, NSW 2052, Australia
E-mail: zi.gu1@unsw.edu.au (Z. Gu)

Dr. L. Zhang
Department of Ultrasound, the First Affiliated Hospital of Chongqing Medical University, Chongqing 400010, China

Dr. S. Cheong
Electron Microscope Unit, University of New South Wales, Sydney, NSW 2052, Australia

Prof. J. J. Gooding
School of Chemistry, ARC Centre of Excellence in Convergent Bio-Nano Science and Technology and Australian Centre for NanoMedicine (ACN), University of New South Wales, Sydney, NSW 2052, Australia

Prof. Y. Chen
State Key Laboratory of High Performance Ceramics and Superfine Microstructure, Shanghai Institute of Ceramics, Chinese Academy of Sciences, Shanghai 200050, China
E-mail: chenyu@mail.sic.ac.cn (Y. Chen)

A: Experimental Section

1. Materials

All chemicals, including $MgCl_2{\cdot}6H_2O$ (>99.0%), $AlCl_3{\cdot}6H_2O$ (>99%), $FeCl_2$ (98%), NaOH (≥98%), 3,3',5,5'-tetramethylbenzidine (TMB), hydrogen peroxide (H_2O_2, 29.0-32.0%), hydrochloric acid (37%), acetic acid (≥99%), sodium acetate anhydrous (≥99%), Dulbecco's Modified Eagle's Medium (DMEM, high glucose), phosphate buffered saline, 2′,7′-dichlorofluorescein diacetate (DCFH-DA, >97%), fluorescein isothiocyanate isomer (FITC), paraformaldehyde, and 2-(4-amidinophenyl) -6- indolecarbamidine dihydrochloride (DAPI, ≥98%) were purchased from Sigma-Aldrich. Roswell Park Memorial Institute medium (RPMI 1640), penicillin/streptomycin and fetal calf serum (FCS) were purchased from Gibco. The

ProLong® Gold Antifade Mountant with DAPI and alamarBlue® were purchased from Thermo Fisher Scientific. The 5, 5-Dimethyl-1-pyrroline N-oxide (DMPO, ≥98%) and cell counting kit-8 (CCK8) were purchased from ENZO and Donjindo respectively. Phosphonic acid terminated poly(ethylene glycol) was synthesized using a controlled/living radical polymerization according to procedures described previously.[1] Milli-Q water was used in the experiments.

2. Synthesis of ultrathin LDH nanosheets and bulk LDH nanoparticles

The LDH nanosheets were synthesized by a solvent-free bottom-up method. Solution A (10 mL) containing $FeCl_2$ (2 mmoL) and $AlCl_3{\cdot}6H_2O$ (1 mmoL) was mixed with Solution B (10 mL) containing NaOH (6 mmoL) with a constant pH value under nitrogen atmosphere, followed by mixing with Solution C containing phosphonic acid terminated poly (ethylene glycol) (0.2 g PEG, 8000 g mL^{-1}). The mixture was then hydrothermally treated at 100 ^{o}C for 8 h. The PEG/Fe-LDH nanosheets were subsequently obtained, after removing extra salt and unattached PEG via centrifugation. Similarly, the PEG/Mg-LDH was prepared by adopting the aforementioned procedure but replacing $FeCl_2$ with $MgCl_2{\cdot}6H_2O$.

The Fe-LDH nanoparticles were synthesized by mixing Solution A and Solution B at a constant pH value under nitrogen protection for 30 min followed by hydrothermal treatment and centrifugation.

The fluorescein FITC-labelled LDHs were prepared by mixing the as-prepared LDH suspension (10 mL, 4 mg mL^{-1}) with FITC solution (0.2 mL, 25 mM) as described previously, and designated as PEG/Fe-LDH-FITC and Fe-LDH-FITC.

3. Characterizations

X-ray diffraction (XRD) measurements were performed on LDH powder and film samples using a PANalytical expert multipurpose X-ray diffraction system (MPD) instrument operated

at 45 kV and 40 mA and fitted with a CuKα source. A scan rate of 0.01°/min was applied with a step size of 2θ = 0.0260°. Size distribution and zeta potential were measured using a Malvern Zetasizer Nano Series. The polydispersity index (PDI) was used to describe particle size distribution. Fourier transform infrared spectroscopy (FTIR) was carried out on a Bruker IFS 66/S single-beam spectrometer. Spectra were obtained at regular time intervals in the MIR region of 4000 – 400 cm^{-1} at a resolution of 4 cm^{-1} (32 scans). Thermal gravimetric analysis (TGA) was conducted using a TGA Q5000 instrument. Powder samples were placed in a platinum pan and heated in nitrogen atmosphere from room temperature to 800 °C at 20 °C min^{-1}. Transmission electronic microscopy (TEM) and scanning transmission electronic microscopy (STEM) imaging coupled and energy-dispersive X-ray spectroscopy (EDS) elemental analysis were performed on a FEI Tecnai G2 (200 kV) and JEOL JEM-F200 (200 kV) equipped with an annular dark-field detector and a JEOL windowless 100 mm^2 silicon drift X-ray detector. EDS data processing and analysis were carried out using the Thermo Scientific Pathfinder X-ray Microanalysis Software. The morphology of nanoparticles was observed using a Transmission Electronic Microscope (TEM, FEI Tecnai G2) at an accelerating voltage of 200 kV. X-ray photoelectron spectroscopy (XPS) was performed using a Thermo ESCALAB250i spectrometer with a monochromatic X-Ray source (AlKα, 1486.68 eV) operated at a 164 W emission power. XPS spectra were analyzed using Avantage 4.88 software. Electron spin resonance (ESR) spectra were obtained at room temperature in perpendicular mode on a Bruker EMX-8/2.7 spectrometer with the following settings: microwave frequency = 9.773 GHz, microwave power = 0.6325 mW, modulation frequency = 100.00 kHz and modulation amplitude = 2.00 G. DMPO was used as a spin trap.

4. Michaelis-Menten kinetics

TMB assay was conducted to monitor the chromogenic reaction (λ = 650 nm) of LDH/H_2O_2 system. In a HAc-NaAc buffer solution (800 μL), LDH suspension (2 μg mL^{-1}) was mixed with TMB and H_2O_2 at the final concentrations of 800 μM and 1 mM respectively. The absorbance of the mixture was measured on a UV-vis spectrometer (CARY 300, Varian) equipped with a temperature controller. For the kinetic study, the concentrations of H_2O_2 (0.01, 0.05, 0.1, 0.2, 0.5, 1, 1.5, 2, 4, and 20 mM) were varied while the concentrations of TMB (800 μM) remained fixed. The Michaelis-Menten constant was calculated by using Lineweaver-Burk plots of the Michaelis-Menten equation: $\frac{1}{v} = \frac{K_m}{V_m} \times (C + \frac{1}{K_m})$, where v represents the initial velocity, V_{max} the maximum reaction velocity, C the concentration of substrate, and K_m the Michaelis-Menten constant.

5. Biodegradation of PEG/Fe-LDHs

The PEG/Fe-LDH nanosheets were suspended in a dialysis tube (MWCO 3500Da) in the buffer of pH 5, 6.5 or 7.4. An aliquot of leachate (2 mL) was sampled at a certain time point, and 2 mL fresh buffer was then added to replace the extracted solution in the suspension. The iron content in the leachate aliquots were measured on a PerkinElmer OPTIMA 7300 inductively coupled plasma optical emission spectroscopy (ICP-OES). The proportion of iron ions released was calculated as:

$$\text{Released percentage (\%)} = \frac{\sum_{i\text{-}1}^{1} c_{i\text{-}1} + 100\, c_i}{c_0} \times 100\%$$

where $\boldsymbol{i}$ represents the sampling time; $\boldsymbol{c_i}$ (mg/mL) the sample content; $\boldsymbol{c_0}$ (mg/mL) the initial metal content. The leachate (containing released iron ions Fe^{2+} and Fe^{3+}) was also sampled to monitor •OH generation from the released solution *via* ESR.

To collect the releasing suspension, the PEG/Fe-LDHs were directly suspended in the buffers of different pH values, and suspension aliquots containing both released ions and PEG/Fe-

LDH particles were collected for TEM observation and ESR test. The relative ESR intensity = (ESR signal intensity at each time points at different pH values / maximum ESR signal intensity at pH 5.0).

6. Cell Culture

The breast cancer cells (4T1 cells and MCF-7 cells) and fibroblast (Hs27 cells) were cultured in a growth medium (RPMI1640, DMEM and RPMI1640 with glutamine respectively) supplemented with 10% fetal calf serum (FCS), streptomycin (100 mg mL^{-1}) and penicillin (100 units mL^{-1}). The cells were cultured at 37 °C in a humidified atmosphere with 5% CO_2 in air.

7. *In vitro* anti-cancer effect evaluation

The 4T1 cells were seeded (1×10^4 cells in 100 μL RMPI1640 per well, pH 7.4) in 96-well microplates and allowed to adhere overnight. Cell culture media at pH 6.5 was used to simulate the extracellular microenvironment in a solid tumor. Hydrochloric acid was added to the RPMI (pH = 7.4) in order to acidize it to a pH of 6.5. The culture medium was then replaced with fresh RPMI (pH 6.5 or pH 7.4) containing PEG/Fe-LDHs at concentrations of 0, 0.03, 0.15, 0.3, 1.5, 3 and 6 µg mL^{-1}. To test the cell tolerance to H_2O_2, cell culture media containing different concentrations (25, 50, 100, 200, 300, 400 and 500 μM) of H_2O_2 were used. After 24 h incubation, the culture media were replaced by with RPMI media containing 10% CCK-8. After 1 h incubation, cell viability was determined by comparing the absorbance at λ 450 nm to the control group. The absorbance of samples was measured on a microplate reader 5 (FLUOstar, Omega, Germany). The experiments were carried out in triplicate, and the values from each experiment calculated from 6 wells.

8. Intracellular ROS detection

The 4T1 cells were seeded at a density of 1 × 10^5 in RPMI of pH = 7.4 in the φ 15 CLSM-exclusive culture disk and allowed to adhere overnight. After incubation with DCFH-DA (20 μM in FCS-free RPMI) at 37 ^oC in 5% CO_2 for 20 min, the culture medium was replaced with medium of pH 6.5 containing 6 μg mL^{-1} PEG/Fe-LDHs and 100 μM H_2O_2, and incubated for 2 h. The treatments of pure culture medium, PEG/Fe-LDHs only, H_2O_2 only, and PEG/Fe-LDHs and H_2O_2 at pH 7.4 culture medium were adopted as controls. The cells were then washed with PBS for three times, fixed with 4% PFA and stained with DAPI for confocal microscopy. The level of intracellular ROS was evaluated by detecting the fluorescence of DCF (λ_{ex} = 488 nm, λ_{em} = 525 nm for DCFH-DA; λ_{ex} = 358 nm, λ_{em} = 461 nm for DAPI) with a confocal laser scanning microscopy (FV 1000, Olympus, Japan).

9. Cellular uptake

MCF-7 cells were seeded in a 24-well plate with coverslips at a density of 5×10^4 cells per well. At semi-confluency, culture medium was replaced with medium containing PEG/LDH-FITC or LDH-FITC (10 μg mL^{-1}). After a given period of time (1, 24, 48 and 72h), cells were washed with PBS three times and fixed with 4% PFA, and the coverslip mounted on glass slides with ProLong® Gold Antifade Mountant with DAPI. The cellular uptake efficiency of PEG/LDH-FTIC and LDH-FITC was observed using Olympus BX53 fluorescent microscope coupled with X-Cite 120Q lamp (Lumen Dynamics).

The percentage of fluorescent cells was quantified by flow cytometry. MCF-7 cells were seeded in a 6-well plate. After 24 h incubation, culture medium was replaced with medium containing 10 μg mL^{-1} PEG/LDH-FITC or LDH-FITC. After a certain period of time (1, 24, 48 and 72 h), the cells were washed three times, collected and fixed with 4% PFA. All samples were monitored on the ACCURI C6 cytometer (BD California, USA).

10. *In vitro* toxicity study

The Hs27 fibroblast cells were seeded (1×10^4 cells in 100 μL RMPI1640 with glutamine per well) in 96-well microplates. When the culture reached 50% confluency, the culture medium was then replaced with fresh RPMI1640 with glutamine containing PEG/Fe-LDHs at concentrations of 0, 0.03, 0.15, 0.3, 1.5, 3, 6 and 12 μg mL^{-1}. After 24 h incubation, the culture media were replaced by with RPMI media containing 10% alamarBlue and incubated for 1 h. Cell viability was determined by comparing the absorbance at λ 570 nm to the control group. The absorbance of samples was measured on a microplate reader 5 (FLUOstar, Omega, Germany). The experiments were carried out in triplicate, and the values from each experiment calculated from 3 wells.

11. *In vivo* toxicity study

All animal experiment operations were performed with approval of the Aminal Ethics Committees of University of New South Wales and Chongqing Medical University. The 7-week female Balb/c mice (~ 20 g) were intravenously injected with PEG/Fe-LDH saline solution (150 μL, 10, 40 and 100 mg kg^{-1} Fe) with the same volume of saline as the control, the body weight of mice was measured every two days (n = 5 for each group). At 30 days, blood was collected for hematological and biomedical indexes analysis. Mice were then sacrificed to collect their major organs (heart, liver, spleen, lung, and kidney) in a 10% formalin solution for histopathology analysis using a typical hematoxylin and eosin (H&E) staining assay.

12. *In vivo* anti-cancer effect evaluation

Xenografted tumors were generated in female Balb/c mice at 7 weeks of age (~ 20 g) by subcutaneously injecting 1×10^6 4T1 cells suspended in PBS (100 μL) into the mouse rear leg. Once the tumor reached a volume of 100 mm^3, 5 mice per group were randomly allocated for different treatments. As a proof-of-principle assessment of Fe-LDH as a catalytic therapeutic

agent, 50 μL of PEG/Fe-LDH saline solution (15 mg kg^{-1} Fe) was injected intratumoral into the tumor, with the control groups undergoing an intratumoral injection of saline solution (50 μL) or PEG/Mg-LDH saline solution (50 μL, 15 mg kg^{-1} Mg). Tumors were measured every two days with a digital caliper. The tumor volumes (V) were calculated as $V = L \times W^2 / 2$, and normalized to the initial volume (V_0) to obtain the relative tumor volume (V/V_0). The pathological tissue sections of tumors were collected in 12 h post-treatment for H&E staining assay. Mice were euthanized once tumor volume reached 1000 mm^3.

13. Statistical Analysis

Quantitative data are presented as mean ± SD and analyzed by one-way ANOVA with TUKEY post-tests using GraphPad Prism software; a *P*-value < 0.05 was considered statistically significant (*$P < 0.05$, **$P < 0.01$, and ***$P < 0.001$).

B: Supplementary Figures

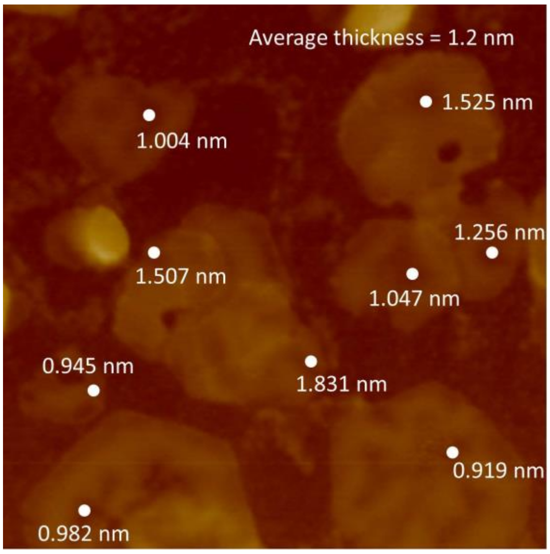

Figure S1. Thickness of 9 thin film spots of PEG/Fe-LDHs measured from AFM image.

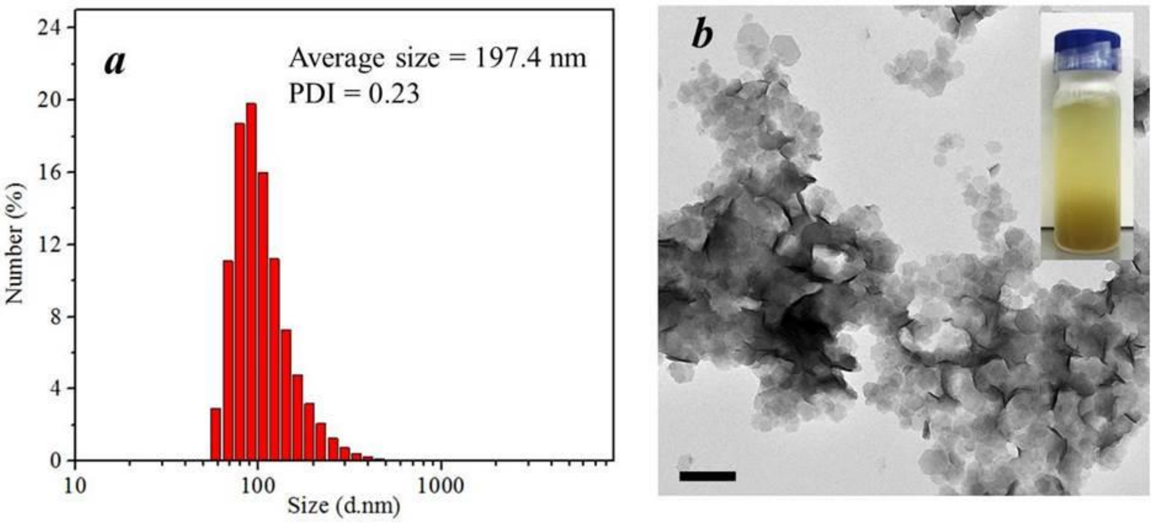

Figure S2. (a) Size distribution of PEG/Fe-LDHs in saline measured by DLS. (b) TEM image of Fe-LDH, scale bar 500 nm (insert digital photo of the Fe-LDH aqueous solution).

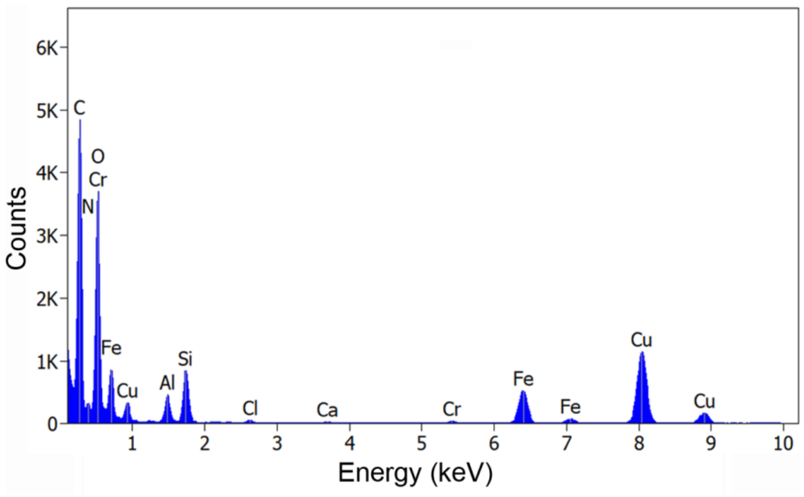

Figure S3. Energy-dispersive X-ray spectrum of the PEG/Fe-LDH nanosheet.

Comparison of TMB assay with ESR spectroscopy by using ammonium iron (II) sulfate ($(NH_4)_2$ Fe $(SO_4)_2$) as a Fenton catalyst. The ESR is a direct approach to monitor the hydroxyl radical generation and thus used to reveal the pH-responsive nature of the PEG/Fe-LDH catalyst. Meanwhile, the TMB assay measures the oxidation capacity of the product generated from the Fenton reaction via a colorimetric reaction below, which was used to evaluate the catalytic efficiency of the PEG/Fe-LDH catalyst. Owing to the acidity-mediated nature of its colorimetric chemical reaction, the TMB approach is not suitable to quantitatively study the pH-responsive catalytic activity of the Fenton catalyst. The insensitivity of TMB assay at pH>6 was further demonstrated by using $(NH_4)_2$ Fe $(SO_4)_2$ as a Fenton catalyst. As shown in the figures below, with the addition of H_2O_2 in the $(NH_4)_2Fe(SO_4)_2$ solution containing free ferrous ions, the TMB assay gave much stronger absorbance at pH 5 than that at pH 6.5 and 7.4 (**Figure S4a**). However, a typical 1:2:2:1 signal of the DMPO•OH adduct was clearly shown in the ESR spectra with the similar signal intensity at pH 5, 6.5 and 7.4 (**Figure S4b**).

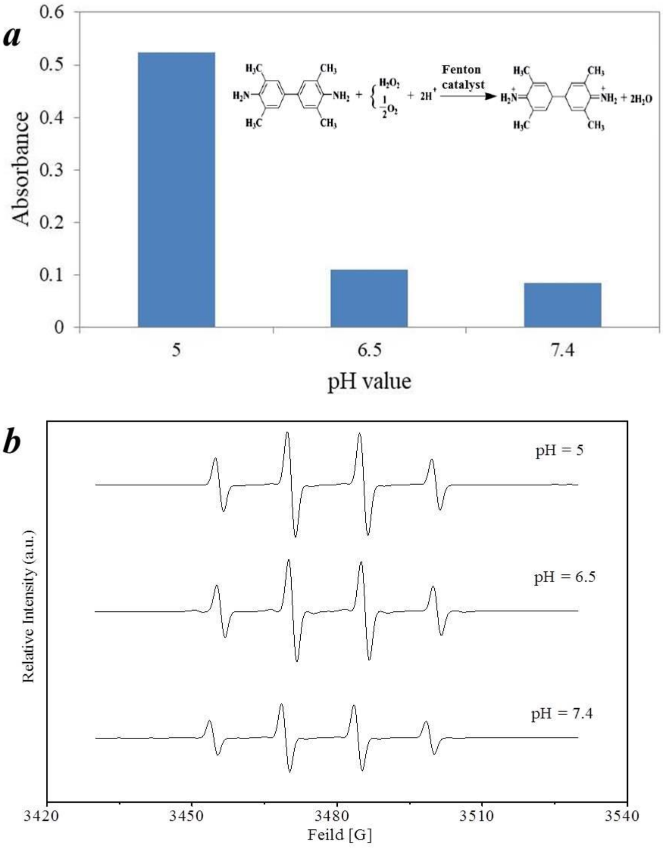

Figure S4. Catalytic performance of ammonium iron (II) sulfate ($(NH_4)_2Fe(SO_4)_2$) measured by TMB assay (a; Insert shows the colorimetric chemical reaction of TMB assay) and ESR spectroscopy (b).

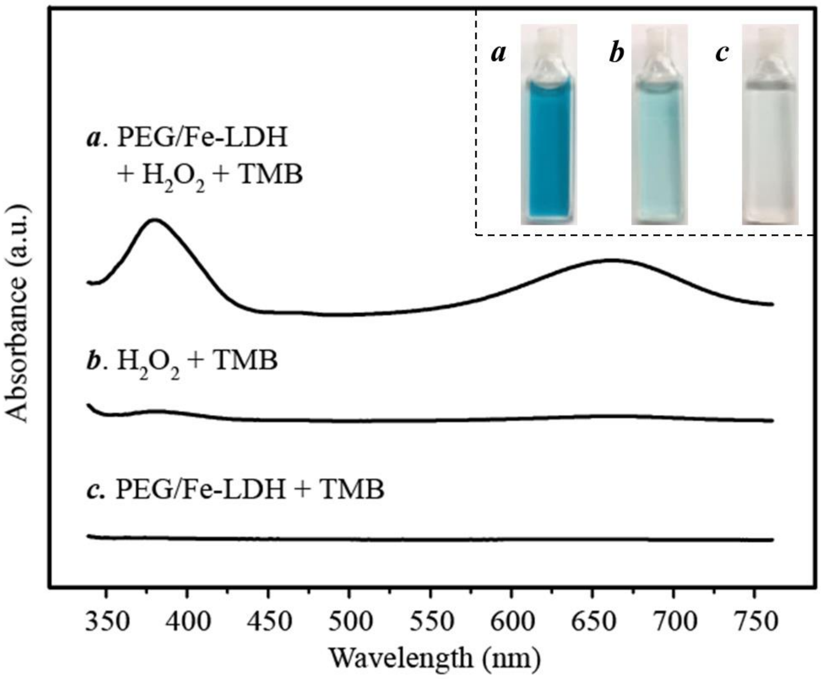

Figure S5. UV-Vis absorbance spectra of TMB colorimetric reaction (Inset shows the digital photos of reactions with different components).

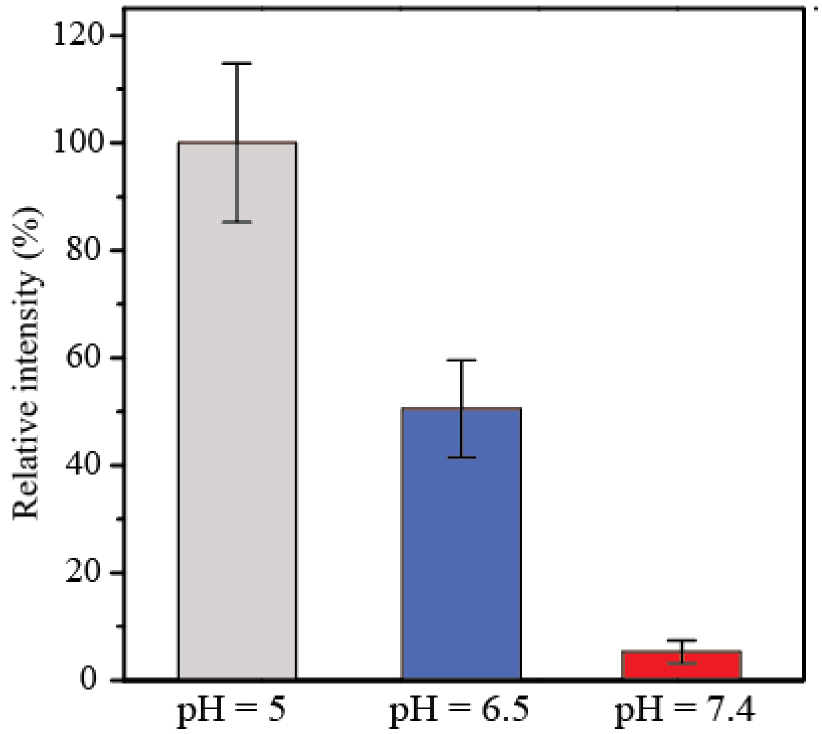

Figure S6. Relative intensity of hydroxyl radical generation derived from ESR spectra.

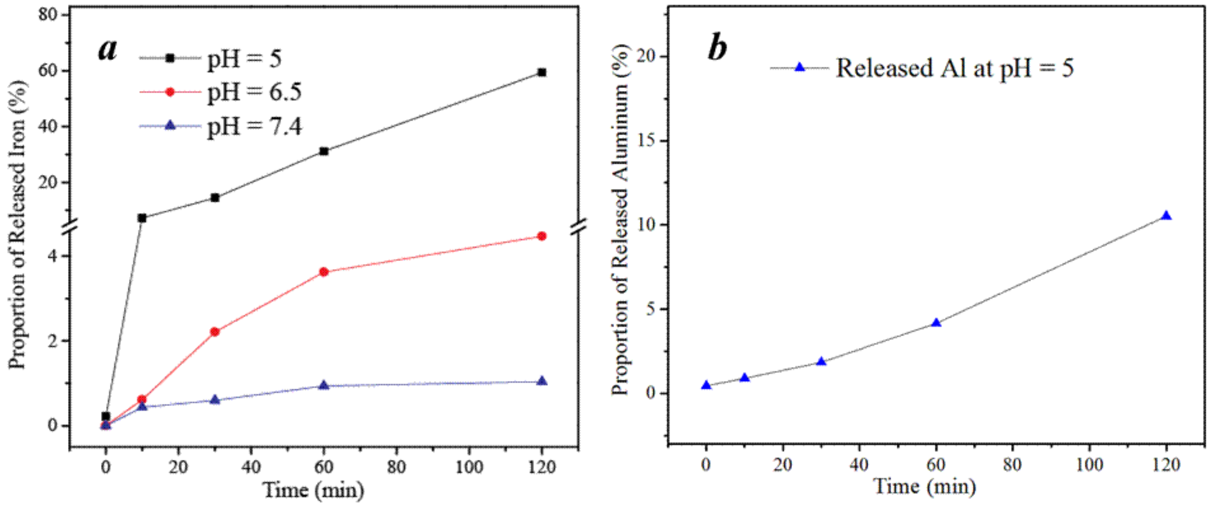

Figure S7. Release profiles of iron (a) and aluminum (b) from PEG/Fe-LDHs under different pH values. The released aluminum at pH 6.5 and pH 7.4 is not detectable.

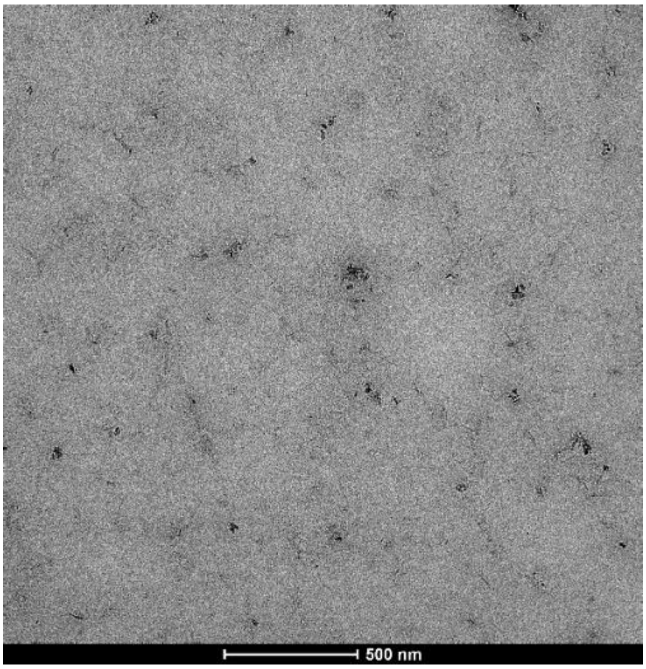

Figure S8. TEM image of PEG/Fe-LDHs after 4-h dissolution in pH 5.0 buffer.

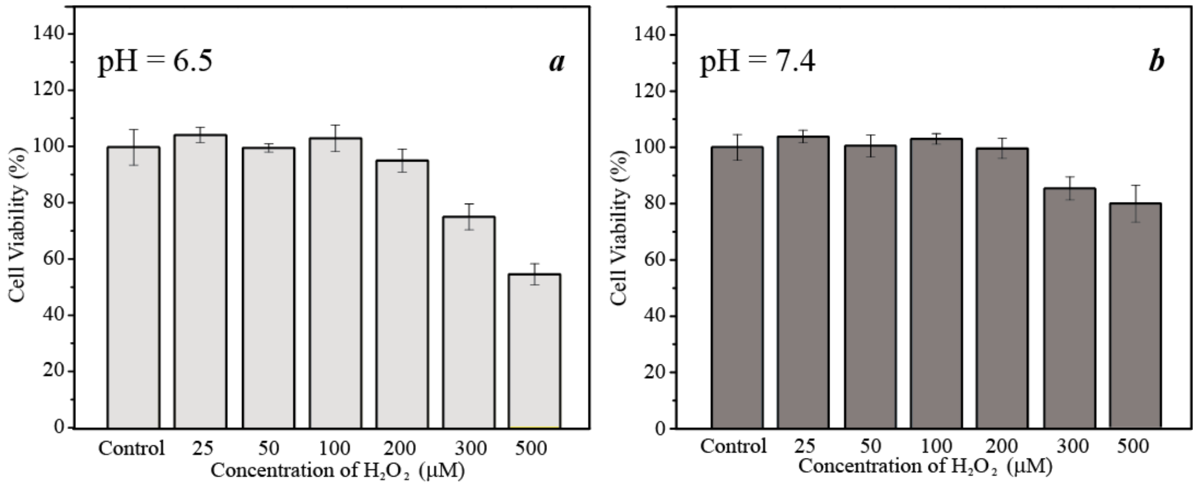

Figure S9. Tolerance of 4T1 cells with various H_2O_2 concentrations at pH 6.5 (***a***) and pH 7.4 (***b***).

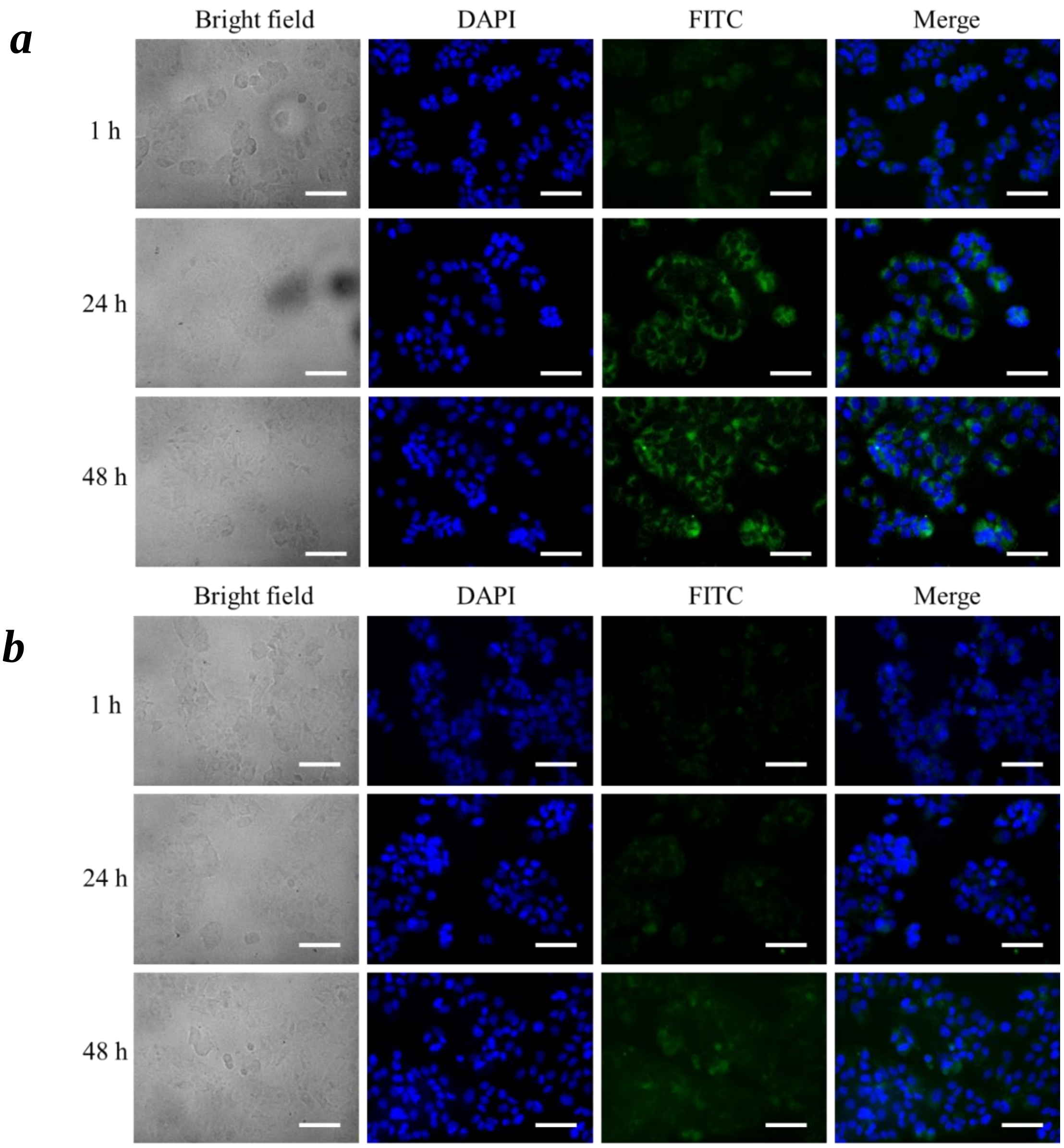

Figure S10. Fluorescence microscopy images of MCF-7 cells treated with (***a***) PEG/Fe-LDH-FITC and (***b***) Fe-LDH-FITC; scale bar 100 µm.

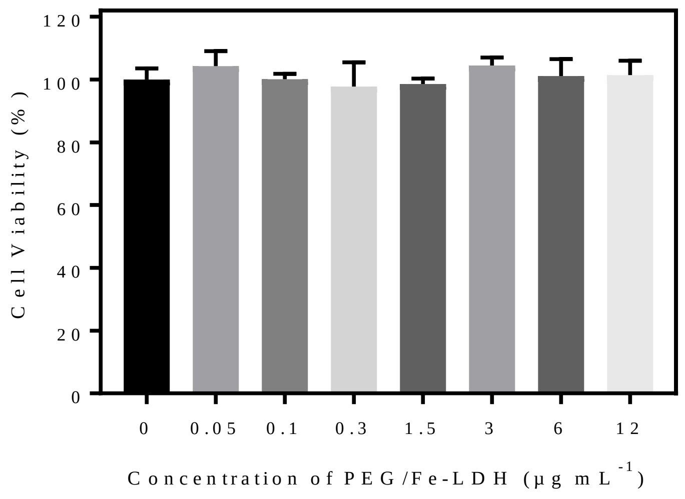

Figure S11. Cell viability of Hs27 fibroblast cells treated with the different concentrations of PEG/Fe-LDH.

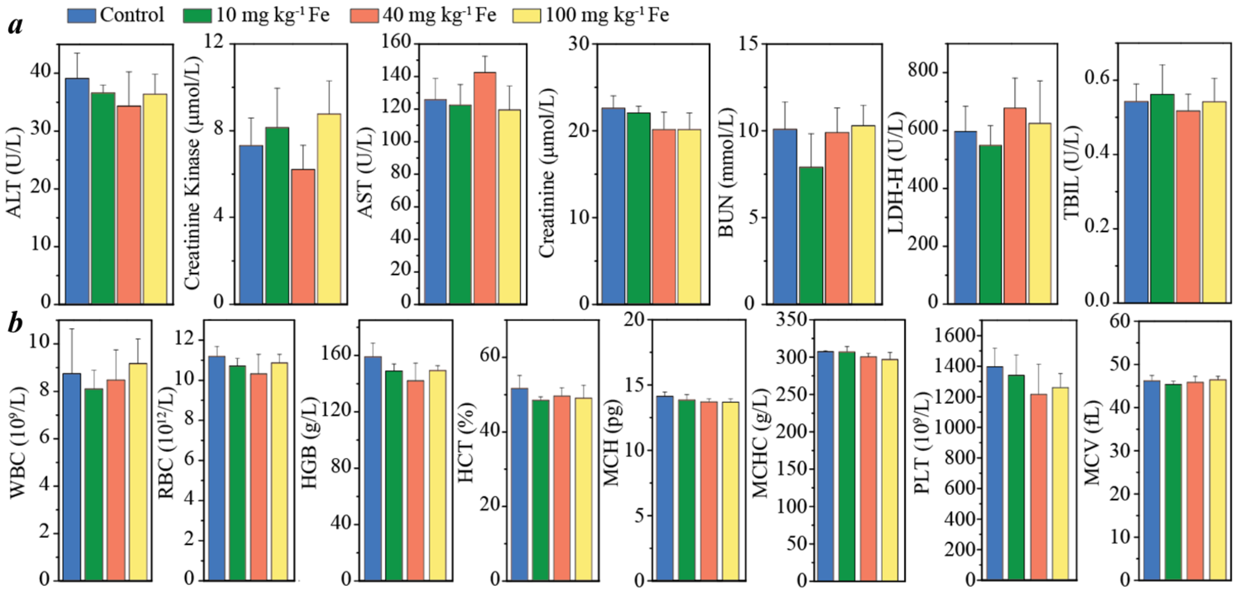

Figure S12. (***a***) Biochemical blood indexes of the Balb/c mice with intravenous injection of PEG/Fe-LDHs of 10, 40 and 100 mg kg^{-1} Fe. (***b***) Hematological indexes of the Balb/c mice treated PEG/Fe-LDHs of 10, 40 and 100 mg kg^{-1} Fe.

References

[1] N. N. M. Adnan, Y. Y. Cheng, N. M. N. Ong, T. T. Kamaruddin, E. Rozlan, T. W. Schmidt, H. T. T. Duong, C. Boyer, Polym. Chem. 2016, 7, 2888; H. T. Duong, K. Jung, S. K. Kutty, S. Agustina, N. N. Adnan, J. S. Basuki, N. Kumar, T. P. Davis, N. Barraud, C. Boyer, *Biomacromolecules* **2014**, 15, 2583.
